# Analyzing the Impact of Four Cognitive Constructs on NVIQ Test Performance: Implications for Children with Neurodevelopmental Disorders

**DOI:** 10.1044/2025_LSHSS-24-00056

**Published:** 2025-05-14

**Authors:** Hope Sparks Lancaster, Erin Smolak, Alice Milne, Katherine R. Gordon, Samantha N. Emerson, Claire Selin

**Affiliations:** 1 Center for Childhood Deafness Language and Learning, Boys Town National Research Hospital, Omaha NE; 2 Department of Communication Sciences and Disorders, University of South Carolina, Columbia SC; 3 Department of Psychology, Lancaster University, Lancaster, UK; 4 Ear Institute, University College London, London, UK; 5 Training, Learning, and Readiness, Aptima, Inc., Woburn MA

**Keywords:** nonverbal intelligence, developmental language disorder, ADHD, DLD

## Abstract

**Purpose:**

Children with neurodevelopmental disorders historically exhibit lower and more variable nonverbal intelligence (NVIQ) scores compared to their typically developing peers. We hypothesize that the intrinsic characteristics of the tests themselves, particularly the cognitive constructs they assess, may account for both the lower scores and variability across tests and over time. Using a qualitative content analysis approach, we examined the extent to which key cognitive constructs are engaged in NVIQ tests and how these constructs compare across different tests.

**Methods:**

Current editions of seven NVIQ tests were selected based on their relevance in clinical and research settings. Qualitative coding of constructs was developed iteratively by speech-language pathologists (SLPs) and researchers. The codes focused on cognitive domains most affected in highly prevalent neurodevelopmental conditions, including attention, receptive language, statistical learning, and working memory.

**Results:**

We identified multiple sub-features for our constructs of interest. Using this coding framework, we found that NVIQ tests qualitatively differ in the extent to which these four constructs influence test performance.

**Conclusions:**

Our findings suggest that understanding the impact of cognitive constructs on NVIQ tests can help explain why children with neurodevelopmental disorders exhibit lower and more unstable NVIQ scores compared to their peers. We provide recommendations for the use of NVIQ tests with neurodevelopmental disorder populations and encourage researchers and clinicians in speech and hearing sciences and psychology to use our results to inform test interpretation and selection.

Children with neurodevelopmental disorders often score lower and show more variability in performance on nonverbal intelligence tests (NVIQ) than their neurotypical peers (Gallinat & Spaulding, 2014; Plante, 1998). Variability in test performance between individuals and within individuals across tests and over time is particularly notable in conditions like developmental language disorder (DLD) (Botting, 2005; Cole et al., 1994; Krassowski & Plante, 1997; Miller & Gilbert, 2008). While past researchers have argued that lower and unstable NVIQ scores in children with neurodevelopmental disorders are part of the phenotypes, we argue that the test constructs artificially deflate nonverbal IQ scores due to demands on sustained attention, language capacity, or familiarity with test items. For example, verbal skills could support performance on NVIQ tasks; indeed, Durant et al. (2019) noted the impact of verbal skills on NVIQ scores in bilingual children, albeit unequally across different tests. Furthermore, NVIQ tests that use familiar objects as test items (e.g., apples), inherently tied to language, may inadvertently advantage children with typical language skills (e.g., Sapir-Whorf hypothesis, Gerrig & Banaji, 1994). The outcomes of NVIQ testing are crucial as they can influence the eligibility category under which a child receives services and thus the types of services available to them. Therefore, understanding the cognitive constructs assessed by these tests is essential. Our study aims to identify the degree to which key cognitive constructs relevant to neurodevelopmental disorders are required on NVIQ tests. Guided by our team’s expertise in attention and language impairments, these results will help improve the interpretability and applicability of NVIQ test results.

The stated purpose of NVIQ assessments is to measure intelligence without relying on language processing. Currently, NVIQ tests serve various purposes across different settings. In clinical contexts, these tests help determine under which special education category to provide services and assist in designing treatment plans by highlighting a child’s strengths and needs. In schools, Individualized Education Program (IEP) multidisciplinary teams, including educational psychologists and speech-language pathologists (SLPs), use NVIQ test results for eligibility verification decisions, goal setting, intervention planning, and family counseling. For example, in Nebraska, intellectual ability is one of four areas considered when determining if a child qualifies for services under Speech/Language Impairment (92 NAC 51.006) or a different eligibility category (e.g., Intellectual Disability 006.04G; Nebraska Department of Education, Office of Special Education, 2021). In research, NVIQ tests are frequently used to determine study eligibility, classify children, and characterize samples (cf. Ebert & Lee, 2024; Gallinat & Spaulding, 2014). Researchers also use NVIQ scores as covariates in regression analyses to account for variance related to cognitive abilities (cf. Dennis et al., 2009; Elbert & Lee, 2024). Thus, the selection of NVIQ tests can influence research outcomes, especially if studies differ in using NVIQ as inclusion/exclusion criteria for children with specific neurodevelopmental profiles. This, in turn, influences how we develop theories and understand neurodevelopmental disorders.

Regardless of the application, it is imperative to understand exactly what NVIQ tests measure. Recent discussions have highlighted the need for precision in identifying these constructs (see Strand et al., 2020, p. 176). This precision is vital as NVIQ tests serve as proxies for the cognitive constructs they aim to measure. Test manuals offer guidelines on administration, psychometric properties, and development of NVIQ tests. These manuals detail the various cognitive abilities the test developers sought to measure, often within a specified theoretical framework. For example, Wechsler tests are based primarily on the Cattell-Horn-Carroll theoretical framework (e.g., Wechsler, 2012, p. 1). Other tests, like the Test of Nonverbal Intelligence (Brown et al., 2010), do not specify any constructs, implying that these tests are “construct free.” However, no NVIQ test is construct free. Typically, test manuals discuss the impact of specific constructs only when a task directly aims to assess them, such as the impact of working memory on tasks like digit span and picture span on Wechsler tests, but they do not discuss the potential impact of other constructs such as statistical learning (Schapiro & Turk-Browne, 2015).

Test reviews can also be used to understand the cognitive constructs on NVIQ tests while comparing between tests. For example, DeThorne and Schaefer (2004) compared 16 NVIQ tests on administrative details, psychometric properties, and cognitive abilities tested within the Cattell-Horn-Carroll framework. This valuable resource has helped many educational psychologists and SLPs better understand NVIQ tests. However, to our knowledge, there are no resources that compare NVIQ tests based on key constructs relevant to neurodevelopmental disorder profiles (e.g., attention). Clinicians and researchers working with children with neurodevelopmental disorders may be interested in understanding the potential impact of cognitive constructs such as attention, receptive language, statistical learning, and working memory.

There is a complex relationship between neurodevelopmental profiles, such as developmental language disorder (DLD) and attention deficit hyperactivity disorder (ADHD), and the cognitive constructs of attention, receptive language knowledge, statistical learning, and working memory (e.g., Blom & Boerma, 2020; Smolak et al., 2020). Critically, children with neurodevelopmental disorders, as a group, often perform lower than typically developing peers on tests of attention, receptive language knowledge, statistical learning, and working memory skills (Alloway & Gathercole, 2006; Duinmeijer et al., 2012; Ebert & Kohnert, 2011; Smolak et al., 2020; Saffran, 2018). NVIQ tests can place high demands on attention in various ways, including timed tasks. Research has found that, on average, children with language needs have relatively lower attention regulation skills compared to peers with typical language (Duinmeijer et al., 2012; Ebert & Kohnert, 2011; Smolak et al., 2020).

Language knowledge, especially receptive, can also impact NVIQ testing. Research has shown that performance on NVIQ tests is verbally mediated (Durant et al., 2019a). Any use of verbal instructions places demands on children’s receptive language. Furthermore, NVIQ tasks vary in the extent to which children can utilize language knowledge and language strategies to solve tasks. For children with neurodevelopmental disorders, NVIQ performance scores may be suppressed because they are less able to use language-based strategies on these tasks compared to typically developing peers. Statistical learning is tapped on several NVIQ tests through matrix reasoning or pattern completion tasks. Deficits in statistical learning have been documented in several neurodevelopmental disorders, including DLD and ADHD (cf. Saffran, 2018). Thus, NVIQ test scores could, once again, be artificially lowered for children with neurodevelopmental disorders due to statistical learning demands.

Lastly, working memory is frequently tested on NVIQ tests. Wechsler tests generally include at least one working memory task, although other tests may place demands on working memory by requiring a child to maintain and manipulate visual information to complete the task. Some research indicates that working memory capacity is reduced in children with various neurodevelopmental profiles (Alloway & Gathercole, 2006). If reduced working memory capacity is a feature of neurodevelopmental disorders, this limitation may result in lower NVIQ scores compared to individuals without neurodevelopmental disorders. Therefore, we posit that the impact of these constructs explains why the NVIQ scores of children with neurodevelopmental disorders can vary substantially across tests, especially considering the historical findings that neurodevelopmental groups score lower on NVIQ tests (e.g., DLD; Gallinat & Spaulding, 2014).

## Purpose

NVIQ tests are commonly used with children who have neurodevelopmental disorders. Research has often compared these children’s performance to that of neurotypical peers to document and theorize differences (e.g., Botting, 2005; Karmiloff-Smith, 1998; Krassowski & Plante, 1997; Thomas & Karmiloff-Smith, 2002), while test reviews have primarily focused on broad theoretical frameworks, standardization, and psychometric properties (cf. DeThorne & Schaefer, 2004). Currently, there is no comprehensive resource that compares NVIQ tests in relation to key cognitive constructs for neurodevelopmental disorders and the degree to which these constructs may impact measurement. This gap impedes clinicians’ and researchers’ ability to make nuanced NVIQ test comparisons, often resulting in test selection driven by administrative factors. Furthermore, understanding these constructs is crucial for comparing outcomes across research studies, particularly when NVIQ is used as an exclusionary criterion, as it can influence which children are included or excluded from a study. To improve test selection in clinical and research settings, a resource comparing NVIQ tests based on the constructs they assess is essential.

We addressed this gap by conducting a qualitative content analysis to assess the relative degree of attention, receptive language, statistical learning, and working memory for seven frequently used NVIQ tests. This project arose from a weekly meeting focused on DLD. Additionally, select members of the team were particularly interested in ADHD and DLD, either due to lived experience (author HSL) and/or programmatic lines of investigation (authors AM, ES, SE, KG, HSL, CS). Thus, this qualitative content analysis focused specifically on constructs significantly impacted in children with ADHD or DLD. The methodology, results, and conclusions were informed and interpreted within the team’s research and clinical expertise in children with these conditions.

## Methods

### Test Selection

We selected NVIQ tests based on the Gallinat and Spaulding (2014) meta-analysis and input from practicing SLPs. To reflect the tools currently available to clinicians and researchers, we restricted our analysis to current editions (e.g., the Wechsler Intelligence Scales for Children 4th edition instead of the 3rd edition) and excluded all out-of-print or discontinued tests.

### Developing the Coding Scheme

**Figure 1:**
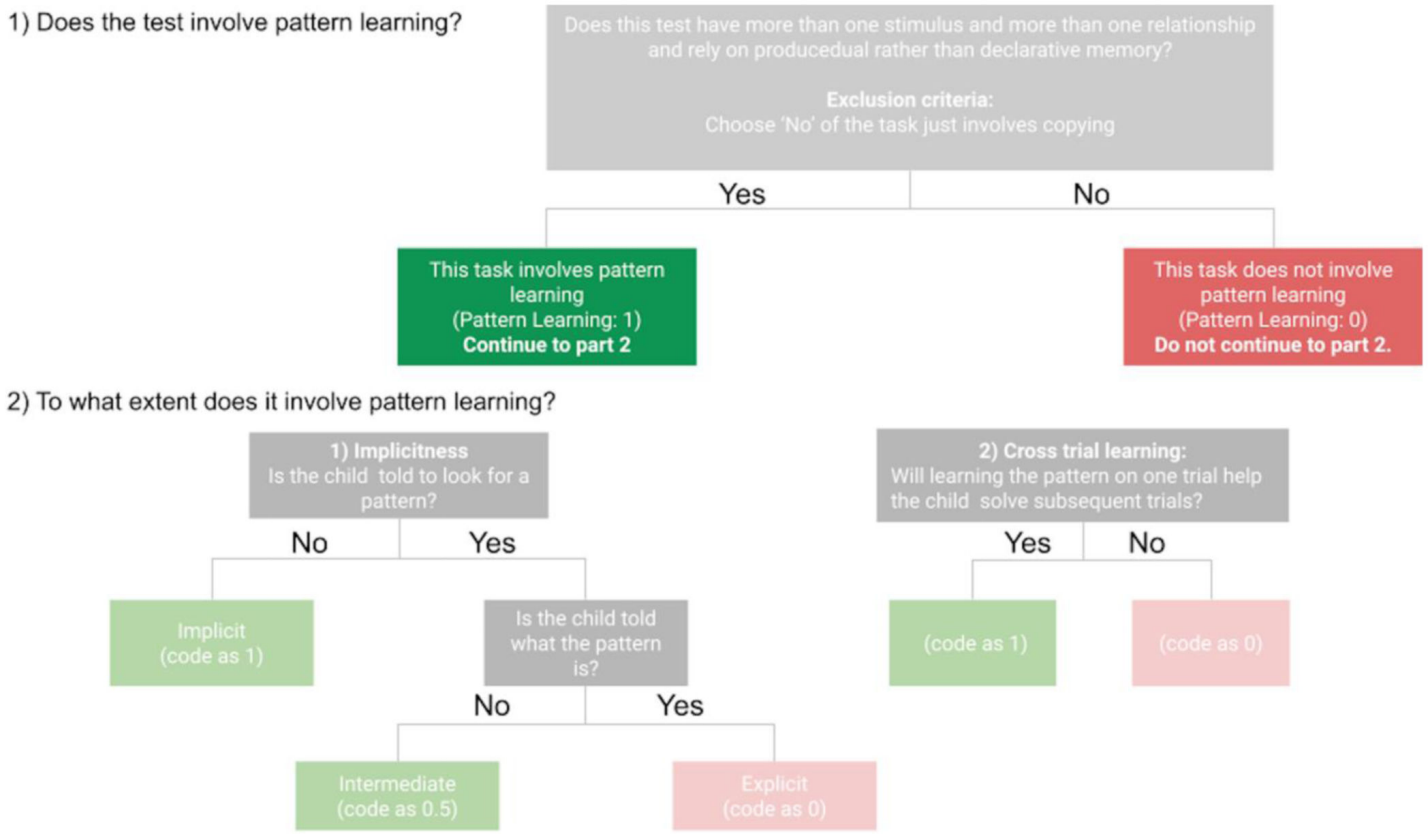
Flow chart for scoring the sub-features related to statistical learning.

We used an iterative process to develop our coding scheme, with final codes and operational definitions provided in Table 1. Our coding team consisted of seven researchers, six holding Ph.D.s in psychology or speech and hearing sciences, and one with a master’s degree

in speech-language pathology. We adopted a combined deductive and inductive content analysis approach (cf. Bengtsson, 2016; Elo & Kyngas, 2008). This approach allowed us to start with pre-defined categories (deductive) while identifying new categories and codes during coding development (inductive). The coding development process involved the following steps:

1. Over several weekly meetings, the team discussed concerns about how receptive language and statistical learning could affect performance on NVIQ tests. These discussions led to a decision to qualitatively analyze seven NVIQ tests for three broad constructs: receptive language, statistical learning, and working memory, which aligned with the expertise of team members.
2. Two coders delineated sub-features and coding schemes for these three constructs, such as verbal instructions for receptive language.
3. The coders developed operational definitions for each sub-feature and initially coded the tests, noting effective and ineffective elements. They also identified aspects of the tests not accounted for in the initial codes.
4. The full research team discussed and refined the coding scheme. During these discussions, the team expanded the scope of codes to include attention and revised sub-features for statistical learning.
5. The coders implemented the revised scheme and made further observations.
6. Additional team discussions led to further revisions of operational definitions and sub-features, especially for statistical learning.
7. Tests were recoded using the updated scheme.
8. External feedback was sought from two researchers with Ph.D.s in psychology to ensure completeness and accuracy of the coding concepts. This feedback led to the inclusion of more detailed codes for attention, consideration of discontinue rules, and combining sub-features for statistical learning.
9. The team created and revised a flow chart for statistical learning codes, shown in Figure 1.
10. During peer review, reviewers suggested changes to our qualitative codes, including adding codes for manipulatives (cf. DeThorne & Schaefer, 2004) and response type. Peer review also requested further clarification in the definitions for attention, statistical learning, and working memory codes.

### Coding and Reliability

All coding was conducted simultaneously by coders, enabling real-time discussion and clarification, ensuring the final codes represented consensus between the two coders, resulting in 100% agreement. To synthesize the coded data, we scored each subtest, averaged these scores, and assigned descriptive ranks to quantify the role of each construct within an NVIQ test. The ranking descriptors used were as follows: None = < .25, Low = .25 to .49, Moderate = .50 to .74, High = .75 to .99, and Very High = 1.

## Results

### Description of NVIQ Tests

The NVIQ tests included in our analysis were: Test of Nonverbal Intelligence - 4th edition (TONI; Brown et al., 2010), Raven’s Progressive Matrices (Raven & Raven, 2003), Wechsler Abbreviated Scales of Intelligence (WASI-2; Wechsler, 2011), Wechsler Intelligence Scales for Children (WISC-V; Wechsler, 2014), Wechsler Preschool and Primary Scales of Intelligence (WPPSI-IV; Wechsler, 2012), Leiter International Performance Scale (Leiter-3; Roid et al., 2013), and Kaufman Brief Intelligence Test (KBIT-2; Kaufman & Kaufman, 2004). Full citations are provided in Supplemental References.

We compared administration details of the NVIQ tests using six general codes: number of subtests, feedback provided, estimated administration time, estimated number of administered items, use of manipulatives, and response format. Based on these codes, the seven tests were broadly similar in administration details. Three tests had one subtest [range = 1 to 6] and took an estimated 20 minutes to administer [range = 15 to 40]. Individual subtests ranged from 12 to 75 potential items (Supplemental Table S1). All seven tests provided feedback on practice items. Four of the seven tests used manipulatives (e.g., blocks, foam tiles), requiring action-based responses (e.g., placing blocks on a table). Additionally, six tests allowed children to respond either nonverbally (e.g., pointing) or verbally (e.g., labeling). Table 2 summarizes descriptive test information and aggregate scores by coded constructs.

### Qualitative Coding

We identified four relevant cognitive constructs from the literature on neurodevelopmental disorders: attention, receptive language knowledge, statistical learning, and working memory. Compared to children with typical development, children with DLD and ADHD exhibit both lower overall performance and high variability in these skills (Alloway & Gathercole, 2006; Smolak et al., 2020; Saffran, 2018). For each construct, we coded two or three sub-features (Table 1). Across constructs, the NVIQ tests varied in the presence and relative degree of these sub-features (none to very high). Aggregate scores across subtests are shown in Table 2, with detailed scores for each subtest provided in Supplemental Table S1.

#### Attention

The system of attention is theorized to consist of three networks: alerting (arousal to stimuli or vigilance), orienting (aligning to the source of sensory input), and executive attention (monitoring and resolving conflict) (Petersen & Posner, 2012; Posner & Rothbart, 2007). Based on this theoretical framework, we coded three sub-features: penalizing changes in phasic attention (i.e., “phasing” in and out; yes/no), timed responses (yes/no), and whether sustained attention was required (yes/no).

Alertness is a state of vigilance and preparation during task performance. Tonic alertness requires sustained vigilance to task goals, while phasic changes in arousal/alertness moment-to-moment can negatively impact task performance (Esterman & Rothlein, 2019; Petersen & Posner, 2012). For example, fluctuations in attention could cause a child to prematurely reach ceiling using a consecutive discontinue rule, whereas a cumulative discontinue rule would allow for phasic changes in alertness. Similarly, performance on timed tasks would be more negatively impacted by phasic changes in alertness compared to non-timed tasks. Finally, sustained attention requires vigilance over an extended period of time and may involve aspects of both tonic alerting and orienting (Tang et al., 2015). Vigilance decrements over time (due to disengagement or depletion of attentional resources) can result in performance deficits.

The NVIQ tests ranged from little to no attention demands to moderate attention demands. Only the Wechsler tests (WASI, WISC-V, WPPSI-IV) required timed responses for at least one subtest. Most NVIQ tests did not require sustained attention. Tests generally allowed children the opportunity to take breaks between subtests, as well as for administrators to redirect the child back to the task at hand. In other words, children were not penalized for a lack of sustained attention. When a test did require sustained attention, it was typically for one or two subtests (e.g., WISC-V Figure Weights).

Most tests penalized children for phasing in and out. Four tests used multiple consecutive failures as their discontinue rule. Therefore, if a child became briefly inattentive, they could prematurely reach ceiling and obtain a score that underestimated their abilities for the explicit constructs measured on individual subtests. The impact of phasic attention was clearest for the Wechsler tests, which had moderate attention demands. These results were driven by two factors: (1) the high number of subtests and (2) the stopping/discontinue rules for these subtests. As demands on phasic attention accumulated over multiple subtests, children with poor attention regulation were more likely to have final scores that underestimated their abilities. For assessments with fewer subtests or alternative stopping rules, the impact of attention performance was less pronounced.

#### Receptive language knowledge

We defined receptive language as the language knowledge stored in a child’s long-term memory that supports comprehension of verbal information, whether provided by another (e.g., verbal instructions) or by the child’s own internal processes (e.g., understanding sub-vocal thoughts). Therefore, we identified two sub-features for receptive language: verbal instructions and possible verbal strategy use.

All NVIQ tests required some level of receptive language (aggregate score range: 0.5–0.83), and five tests had high demands. Three distinct patterns emerged across the tests:

1. Moderate verbal instructions plus verbal strategy use (TONI).
2. High verbal instructions with little opportunity for verbal strategy use (Raven’s Matrix).
3. No verbal instructions with high potential for verbal strategy use (Leiter-3).

For example, the TONI had an aggregate receptive language score of 0.75 (high), driven by its moderate use of verbal instructions (i.e., less than 50 words and no complex syntax; see Table 2 and Supplemental Table S1) and a high verbal strategy score (“Which one of these goes in this box?” page 5, Brown et al., 2010). Although Raven’s Matrix included verbal instructions with subordinate clauses, the highly abstract items reduced the likelihood of using verbal strategies, as children may lack the words to describe the items. In contrast, the Leiter-3 explicitly instructed administrators not to use language, instead providing suggested gestures to demonstrate instructions. Additionally, the Leiter-3 often used familiar items, particularly for younger children. The use of familiar items could increase the likelihood of a child employing verbal strategies to solve problems, as they can rely on verbal labels stored in long-term memory (e.g., “apple”) to support performance.

We also observed that within individual NVIQ tests, receptive language demands varied across subtests. This pattern is most evident in the WISC-V, which has an aggregate receptive language score of 0.83 (high). This high score was primarily influenced by two subtests (visual puzzles and coding), where both verbal instructions and verbal strategy use were scored as high. These subtests featured complex verbal instructions and a high potential for children to use verbal strategies.

#### Statistical learning

Statistical learning coding followed the flow diagram in Figure 1. Our operational definition was based on Frost, Armstrong, and Christiansen’s (2019) definition, which states that statistical learning involves “perceiving and learning any forms of patterning in the environment that are either spatial or temporal in nature” (p. 1130). Accordingly, we identified three sub-features: pattern learning, implicitness, and cross-trial learning.

We first determined whether the subtest contained a pattern learning component and then rated the sub-features of implicitness and cross-trial learning to evaluate how much a child’s ability to respond to regularities influenced their performance. By definition, patterning requires more than one stimulus (an independent stimulus is not a pattern) and more than a single occurrence of events in the stream (a single appearance is not a pattern) (Frost et al., 2019, p. 1130).

The inclusion of implicitness as a sub-feature was informed by literature suggesting that statistical learning and implicit learning “reflect a type of incidental pattern learning (i.e., learning occurring without intention or instruction)” (Conway, 2020, p. 280) and that “[…] statistical learning can occur largely automatically, without intent, without conscious awareness, and that it is often implicit and incidental” (Frost et al., 2019, p. 1145). In our coding, implicitness evaluated whether test instructions explicitly alerted children to the presence of a pattern.

The cross-trial learning sub-feature was derived from procedural learning literature, which describes how “learning occurs on an ongoing basis during multiple trials” (Ullman & Pierpont, 2005, p. 401).

All NVIQ tests included at least one subtest that tapped statistical learning. Regarding pattern learning components, requirements differed more between subtests within a given NVIQ test than between the tests overall. Some subtests did not require pattern learning at all (e.g., block design), while others heavily relied on it (e.g., matrix reasoning). This variability often resulted in intermediate pattern learning requirements at the test level. For example:

- The **WISC-V** included two subtests with high (matrix reasoning) or very high (coding) pattern learning demands, while the other four subtests did not involve pattern learning. As a result, the WISC-V has a low overall pattern learning demand, assuming all six subtests are administered.
- The **Leiter-3** included two subtests with no pattern learning demands (figure ground and form completion) and two subtests with moderate demands (classification and sequential order), resulting in a low overall pattern learning score.

These examples demonstrate the importance of understanding the influence of cognitive constructs at the subtest level as well as at the overall test level.

There was variability in how explicit the instructions were regarding the presence of patterns in subtests requiring pattern learning. The WISC-V coding instructions indicate that each item will be repeated twice (explicit) but do not allow the assessor to highlight co-occurrences (e.g., triangle goes with circle). The TONI and Leiter-3 provide suggested instructions to indicate to the child that a pattern is expected, whereas the minimalistic instructions on the WPPSI-IV do not allow explicit guidance about patterns.

For cross-trial learning, five of the NVIQ tests included subtests with a limited number of patterns that facilitated cross-trial learning. For instance, the **KBIT-2** matrices task contains approximately three patterns, each grouped together. The first 17 trials involve pairs of known objects that go together (e.g., washing machine and shirt, bathtub and person).

In contrast:

- The **Raven’s Matrix** and the **WPPSI-IV** matrix reasoning subtests involve categories of matrices presented randomly throughout the subtest. This design effectively eliminates opportunities for cross-trial learning in these subtests.

#### Working memory

While various models of working memory exist (e.g., Baddeley, 1986; Cowan, 2001), our analysis was agnostic to the precise mechanisms underlying working memory. For this study, working memory was defined as the system by which information is activated in memory, held in short-term storage, and available for processing. Accordingly, we coded for two sub-features: encoding novel information and holding target information in mind.

The KBIT-2 is the only NVIQ test with no working memory demands according to our coding scheme (no novel information, no target holding). By contrast, the Leiter-3, WISC-V, and WPPSI-IV had moderate working memory demands, while the TONI, Raven’s, and WASI-2 relied heavily on working memory due to their use of novel items and the need to hold targets in mind to complete tasks. Only the WPPSI-IV and WISC-V explicitly stated that they were designed to measure working memory; thus, for these tests, the impact of working memory on measurement is intentional. None of the other NVIQ tests indicated an explicit intention to assess working memory.

Most NVIQ subtests do not explicitly assess working memory. However, a child’s ability to hold the target in mind can still influence performance, even when items and answers are presented simultaneously. The WISC-V picture span and WPPSI-IV picture memory subtests are exceptions, as they were designed specifically to assess working memory by removing the target stimulus. Despite this, these subtests do not significantly increase the overall working memory demands of the WISC-V (0.63) or WPPSI-IV (0.50) compared to other NVIQ tests (e.g., TONI 0.75), likely because the other subtests in these tests have minimal working memory demands.

While simultaneous presentation may suggest that items do not need to be held in memory, children who can remember the target and then select the answer without revisiting the target likely have a higher working memory capacity. This ability enables consistent performance across tasks with either simultaneous or non-simultaneous presentations. Conversely, children lacking this ability might perform better on tasks with simultaneous presentations, where activating working memory is less critical. Most tests are designed to encourage the strategic approach of keeping the target in mind while selecting the answer, regardless of their outward presentation.

Our coding for novelty showed greater variability across NVIQ tests than target holding. Most tests included subtests with novel items, such as block design tasks that featured new patterns of colored squares. However, the Leiter-3 and KBIT-2 primarily used familiar items. For example, in the Leiter-3, early items involve recognizable objects like balloons or trees, with more challenging items requiring manipulation of parts of these familiar objects, such as circle segments. Similarly, the KBIT-2’s matrix task included grouped patterns, with the first 17 trials consisting of pairs of commonly associated objects, like a washing machine and a shirt.

## Discussion

NVIQ tests aim to measure general intellectual abilities without bias from receptive or expressive language skills. However, research indicates that individuals with neurodevelopmental disorders often score lower and exhibit more variability on these tests compared to neurotypical individuals. Traditional interpretations of these differences attribute the lower scores to the phenotype of the disorders. In contrast, this study offers an alternative explanation: the cognitive constructs engaged by NVIQ tests may impact performance in children with neurodevelopmental disorders.

Our findings build on prior research by focusing on constructs associated with multiple neurodevelopmental disorders and comparing how common NVIQ tests engage these constructs. Our qualitative content analysis of seven major NVIQ tests highlights two key findings. First, we identified how four cognitive constructs—attention, receptive language, statistical learning, and working memory—are engaged across tests. These constructs, often areas of difficulty for neurodevelopmental disorder populations, include various sub-features (Tables 1 and 2) that could significantly influence performance. Second, we found notable differences in the degree to which these constructs and their sub-features are represented across the tests, as detailed in Table 2 and Supplemental Table S1.

The cognitive construct demands of the NVIQ tests analyzed range from minimal to significant. For example, the Raven’s Progressive Matrices showed no attentional demands, while the KBIT-2 had no working memory requirements within our coding framework. These insights provide an alternative perspective on why children with neurodevelopmental disorders often achieve lower NVIQ scores compared to their peers. While prior studies have examined correlations between specific child characteristics and NVIQ outcomes, our findings emphasize the role of the cognitive constructs these tests assess in influencing performance. For instance, a child with developmental language disorder (DLD) may score lower on NVIQ tests due to receptive language demands, potentially misrepresenting their true cognitive aptitude.

The emphasis on particular cognitive constructs in NVIQ tests may also explain the instability of NVIQ scores over time and across different tests in children with neurodevelopmental disorders. For example, consider a child with DLD assessed with the Leiter-3 at age five and the WISC-V at age nine. Differences in attention, receptive language, and working memory demands between these tests could lead to a significant decline in NVIQ scores, potentially in the range of 10 to 30 points as documented in previous research (e.g., Plante, 1998). This example underscores the importance for clinicians and researchers to understand the cognitive constructs engaged by an NVIQ test when evaluating or interpreting results for children with neurodevelopmental disorders.

Understanding the specific constructs assessed by NVIQ tests is critical for accurately representing the cognitive abilities of children with neurodevelopmental disorders. This knowledge enables more precise interpretations of test results and supports appropriate test selection. This study contributes an important perspective to the discussion surrounding NVIQ testing, urging careful consideration of the cognitive constructs engaged by these tests to ensure fairer and more accurate assessments of intellectual ability in children with neurodevelopmental disorders.

### Implications for Clinical Practice

Many standardized tests (e.g., language, general cognition) assess multiple skills, including some not explicitly identified in the test manual. SLPs should be mindful of the key constructs that could influence test performance. A deeper understanding of NVIQ assessments enables SLPs to make more person-centered decisions regarding eligibility, diagnoses, treatment plans, and family counseling. Given the high-stakes nature of NVIQ assessments, SLPs can collaborate with school psychologists and neuropsychologists to select suitable, appropriate tests (ASHA Assessment and Teaming, n.d.). For example, when language poses a challenge, tests like the Raven’s Progressive Matrices and Leiter-3 are better choices for assessing nonverbal skills with minimal language interference.

Returning to the Nebraska state guidelines, consider a child with suspected DLD being evaluated in Nebraska. As part of the assessment, the multidisciplinary IEP team administers the WISC-V to measure intellectual ability. Children with DLD typically score lower on verbal tasks due to their language-learning profiles. However, our qualitative findings indicate that nonverbal subtests on the WISC-V have high receptive language demands. These demands could lead clinicians to underestimate the child’s nonverbal abilities, potentially resulting in misclassifications such as Intellectual Disability. Using our findings, the SLP on the IEP team could advocate for selecting a different NVIQ test for this child, leading to a more appropriate assessment. Effective interprofessional collaboration in selecting evaluation tools is essential for accurate data interpretation and proper eligibility classifications.

### Implication for Research

Our findings have significant implications for research involving NVIQ tests. These tests are commonly used to determine study eligibility, classify children, match participants by NVIQ, characterize research samples, and control for NVIQ in regression analyses. Concerns regarding the use of NVIQ tests in these contexts have been raised in previous studies (Dennis et al., 2009; Earle et al., 2017; Norbury et al., 2016). Our results suggest that researchers must carefully select NVIQ tests to align with their study objectives, as failure to do so can introduce confounds that affect research results.

The impact of NVIQ test selection on research findings can be illustrated with an example. Consider a study on working memory in children with DLD that uses the TONI to screen participants with a cutoff score of 85, as justified by prior research (e.g., Leonard, 2014). Due to the TONI’s high demands on receptive language and working memory, the participants included in the study would likely have average or above-average working memory skills. This could lead researchers to erroneously conclude that working memory does not differ significantly between children with DLD and those without, thereby affecting the study’s generalizability and theoretical models of working memory’s role in language learning. Alternatively, using tests such as the Raven’s Progressive Matrices, Leiter-3, or KBIT-2 might include a broader range of abilities, reducing confounds and better targeting the construct of interest. This highlights the importance of aligning NVIQ test selection with study goals rather than solely relying on precedent, as test choice can significantly influence study outcomes and interpretations.

### Strengths and Limitations

This study has three main limitations. First, our qualitative coding scheme may not encompass every construct assessed by the NVIQ tests analyzed. We focused on identifying common constructs and their sub-features, particularly those known from previous research to affect NVIQ test performance in children with neurodevelopmental disorders such as ADHD and DLD. However, some NVIQ tests may assess additional constructs that are not common across multiple tests. For example, there is considerable overlap between NVIQ and executive functioning, which we did not include in our coding scheme. We encourage clinicians and researchers to examine the impact of executive functioning on NVIQ test performance in children with neurodevelopment disorders.

Second, we did not analyze every available NVIQ test. Instead, we selected tests that are currently in print and widely used in clinics, schools, and research settings. This selection was informed by clinical SLP input and the tests’ prevalence in research, and the chosen tests have been staples in various settings for over 30 years. Our coding scheme is provided in Table 1, and we invite SLPs and researchers to apply it to other NVIQ tests. To facilitate this, we have established an OSF project for community use of this coding framework, aiming to support the selection and interpretation of NVIQ tests in clinical and research contexts.

Third, we chose to interpret our results with a focus on children with ADHD and DLD. However, there are several other neurodevelopmental disorders that exhibit similar weaknesses in attention, language, statistical learning, and working memory. Clinicians and researchers may wish to reinterpret our findings to consider how these cognitive constructs could impact NVIQ test performance for children with autism spectrum disorder, intellectual disability, or specific learning disabilities (e.g., dyslexia).

## Conclusion

This study explored an alternative explanation for the observed lower and more variable NVIQ scores among children with neurodevelopmental disorders: the influence of attention, receptive language knowledge, statistical learning, and working memory on NVIQ test performance. Building on prior work, we focused on these constructs because they are associated with multiple neurodevelopmental disorders. Although we did not address disorders such as autism in this study, we strongly recommend that researchers with expertise in this area explore the potential implications of these constructs further.

Our multidisciplinary team used qualitative coding to evaluate the extent to which these four key constructs could affect performance on NVIQ measures in children with neurodevelopmental disorders. Of these constructs, only attention and working memory were discussed in test manuals, while the impact of receptive language knowledge and statistical learning was not. These constructs appeared to varying degrees across all the NVIQ tests we analyzed.

It is essential for clinicians and researchers to consider the influence of these constructs when selecting and interpreting NVIQ tests. Our qualitative coding framework, used in combination with test manuals and reviews (e.g., DeThorne & Schaefer, 2004), can serve as a valuable resource to aid in test selection and interpretation.

## Supplementary Material

Supplementary Table S1

## Figures and Tables

**Table 1:** Operational definitions of qualitative codes.

Sub-feature	Operational Definition	Codes	Examples
		**General**	
Required number of subtests	How many tasks are minimally required to get a nonverbal IQ score?	numeric	TONI has 1 task. The WISC has 6 tasks.
Feedback	Does the manual indicate that test administers should give feedback on practice trials?	0 = no 1 = yes	WISC-V states that during practice items, test administers can tell the participant if they were correct or incorrect, in which case the administer should indicate the correct answer option.
Max possible time	What is the maximum possible time a child could spend on this task?	numeric	Per the test manual what is the max allowed or estimated time for a subtest.
Max number of items	What is the maximum number of items on this task/subtest?	numeric	Total count of items in a subtest.
Manipulatives used	Were manipulatives (e.g., blocks, foam squares) used to present items?	0 = no 1 = yes	The TONI-2 does not use manipulatives. Lieter-3 uses a variety of manipulatives including foam shapes and picture cards.
Response type	How were children encouraged or expected to respond to items?	A = action NV = nonverbal V = verbal	Tests code receive more than one code as some tests allowed participants to point (NV) or say a number (V). The use of manipulatives meant a test must have an action score in additional to other response types. Tasks on the Lieter-3 with foam blocks or pictures that needed to be placed received A; whereas tasks that required pointing received a NV code. Thus, the Lieter-3 received A and NV codes as both response types are required. The TONI-2 states that children may point or say the number of the picture, thus it received both NV and V codes as both response types are allowed.
**Attention**			
The system of attention is theorized to consist of the three networks of alerting (arousal to stimuli or vigilance), orienting (aligning to the source of sensory input), and executive attention (monitoring and resolving conflict) (Petersen & Posner, 2012; Posner & Rothbert, 2007).			
Timed response	Are responses timed, or are scores penalized for not finishing in a set amount of time?	0 = not time 1 = timed	Block Design is a timed task for each item.
Phasic attention	Does the task require the child to stay on task for the entire time and does not allow for phasic attention (based on the discontinue rule)?	0 = no 1 = yes	Most Letier-3 stopping rules are based on cumulative failed responses and not consecutive failures. The WISC-V picture span has a stopping rule of 3 consecutive failures.
Sustained attention	Does the task/subtest require sustained attention (based on whether breaks and/or reorienting between items is allowed)?	0 = breaks/reorienting allowed 1 = no breaks or reorienting allowed	On the TONI-2 the child is reoriented before every item with a prompt like, “Now try this one.” The Coding subtest on the WISC-V is based on how many items a child can complete in a set amount of time.
**Statistical Learning**			
We based our operational definition on Frost, Armstrong, and Christiansen’s (2019) definition, which stated that statistical learning deals with “perceiving and learning any forms of patterning in the environment that are either spatial or temporal in nature” (pg 1130).			
Pattern learning	Is there a pattern to be learned (i.e., more than one stimulus and more than one relationship)?	0 = no 1 = yes	Visual Puzzles shows the child a broken-up image (e.g., parts of a beach ball), and he must select an image of the correctly re-assembled parts (e.g., beachball but not the dog).
Implicitness	Are they told to look for a pattern? If so, are they told what the pattern should be?	0 = explicit .5 = intermediate 1 = implicit	TONI-2 uses the word “pattern” so is scored as explicit. Raven’s matrix uses generic language so is scored as 0.5.
Cross-trial learning	Will learning a pattern on one trial help you with a subsequent trial (e.g., “rules” or categories/types of trials)?	0 = no 1 = yes	For example, there are four types of trials for WASI-2 Matrix Reasoning: pattern completion, classification, analogy, and serial reasoning
**Receptive Language Knowledge**			
The language knowledge the child has stored in long-term memory that supports comprehending verbal information, whether that information was provided by another (e.g., verbal instructions) or one’s own self (e.g., understanding one’s sub-vocal thoughts).			
Verbal instructions	Do test administration instructions in the manual include providing the child with a verbal description of how to perform the task?	0 = instructions are completely nonverbal 0.5 = minimal verbal, approximately 50 words and no complex syntax 1 = high verbal, more than 50 words and includes dependent clauses	Lieter-3 gives gesture guidelines and explicitly states not to use language. The TONI provides a brief script and no sentences have more than 1 clause. Raven’s matrices script contains sentences with dependent clauses (e.g., “…because…”.).
Verbal strategy use	Is it possible to use a verbal strategy, and would using a verbal strategy make the task easier?	0 = no 1 = yes	Raven’s Matrix presents highly abstract items which are difficult to describe verbally, which limits the likelihood of using a verbal strategy to support task completion. TONI patterns can be described verbally. A child could use language to identify that they are looking for a piece “containing two straight flat lines” to complete the pattern.
**Working Memory**			
Working memory is the system by which information is activated in memory, is held in some short-term storage, and while it is activated it is available for processing. This definition is designed to be agnostic to existing models of working memory.			
Novelty	Does the task require the child to encode novel information?	1 = items are completely novel 0 = all or some of items are based on known objects	TONI-2 images are not familiar objects, instead they are abstract shapes and shadings. WIPPSI-IV Bug search has the child looking for known insects - ants, spiders, etc.
Hold	Is there a target or referent that needs to be held in mind, and/or does doing so make the item/task easier (i.e., speeded/timed response)?	0 = Nothing is held in mind 0.5 = Holding something in mind could facilitate performance but reference and test items are presented simultaneously 1 = A reference must be held in mind and is not simultaneously presented with test items	The KBIT-2 subtest is an untimed matrix completion task with answer options and target presented on one page. On block design tasks, being able to hold the desired result in short-term memory will speed up response thus allowing the child to receive a higher score. On the WIPPSI Picture Span task, children are presented with a series of pictures and then asked to recall them after the pictures are removed.

*Note*. TONI = Test of Nonverbal Intelligence (Brown, 2003); WASI-2 = Weschler Abbreviated Scales of Intelligence (Wechsler, 2011); WISC-V = Weschler Intelligence Scales for Children (Wechsler, 2014); WPPSI-IV = Weschler Preschool and Primary Scales of Intelligence (Wechsler, 2012); KBIT-2 = Kaufman Brief Intelligence Test (Kaufman & Kaufman, 2004); Leiter-3 = Leiter International Performance Scale (Roid et al., 2013); Raven’s = Raven’s Progressive Matrices (Raven & Raven, 2003).

**Table 2:** Descriptive information and aggregate scores for coded constructs by nonverbal intelligence test.

Test	General Codes						Cognitive Constructs			
	Age Range	# Req. tasks	Feedback^a^	Max possible time (mins)	Manipulatives used^a^	Response type^b^	Attention	Statistical Learning	Receptive Language	Working Memory
Lieter-3	3;0 - 75;11	4	Y	40	Y	A & NV	Low	Low	Moderate	Low
KBIT-2	4;0 - 90;11	1	Y	20	N	NV or V	Low	High	High	None
Ravens	6;0 - 16;11	1	Y	20	N	NV or V	None	Moderate	Moderate	High
TONI-2	6;0 - 89;11	1	Y	20	N	NV or V	Low	Moderate	High	High
WASI-2	6;0 - 90;11	2	Y	15	Y	A & NV or V	Moderate	Low	High	High
WISC-V	6;0 - 16;11	6	Y	40	Y	A & NV or V	Moderate	Low	High	Moderate
WIPPSI- IV	2;6 - 7;7	5	Y	30	Y	A & NV or V	Moderate	Low	High	Moderate

Notes. Aggregate / summary scores were obtained by averaging the scores for sub-features within a construct and across subtests as needed. These scores were then assigned a descriptive term based on the following criteria: None = < .25 Low = .25 to .49, Moderate = .50 to .74, High = .75 to .99, Very high = 1.

a Y = yes; N = no.

b A = action required i.e., placing pictures in order; NV = nonverbal response such as pointing; V = verbal response allowed

## Data Availability

https://osf.io/3vb57/?view_only=f53f2d3f8635491fa696b38fb3664e4a

## References

[R1] Alloway TP, Gathercole SE (2006). Working Memory and Neurodevelopmental Disorders.

[R2] Baddeley A (1986). Working memory.

[R3] Bengtsson M (2016). How to plan and perform a qualitative study using content analysis. NursingPlus Open.

[R4] Blom E, Boerma T (2020). Do children with developmental language disorder (DLD) have difficulties with interference control, visuospatial working memory, and selective attention? Developmental patterns and the role of severity and persistence of DLD. Journal of Speech, Language, and Hearing Research.

[R5] Brown L, Sherbenou RJ, Johnsen SK (2010). Test of Nonverbal Intelligence, Fourth Edition.

[R6] Cole KN, Mills PE, Kelley D (1994). Agreement of assessment profiles used in cognitive referencing. Language, Speech, and Hearing Services in Schools.

[R7] Conway CM (2020). How does the brain learn environmental structure? Ten core principles for understanding the neurocognitive mechanisms of statistical learning. Neuroscience & Biobehavioral Reviews.

[R8] Cowan N (2001). The magical number 4 in short-term memory: A reconsideration of mental storage capacity. Behavioral and Brain Sciences.

[R9] Dennis M, Francis DJ, Cirino PT, Schachar R, Barnes MA, Fletcher JM (2009). Why IQ is not a covariate in cognitive studies of neurodevelopmental disorders. Journal of the International Neuropsychological Society.

[R10] DeThorne LS, Schaefer BA (2004). A guide to child nonverbal IQ measures. American Journal of Speech-Language Pathology.

[R11] DeThorne LS, Watkins RV (2001). Listeners’ perceptions of language use in children. Language, Speech, and Hearing Services in Schools.

[R12] Duinmeijer I, de Jong J, Scheper A (2012). Narrative abilities, memory and attention in children with a specific language impairment. International Journal of Language & Communication Disorders.

[R13] Durant K, Peña E, Peña A, Bedore LM, Muñoz MR (2019a). Not all nonverbal tasks are equally nonverbal: Comparing two tasks in bilingual kindergartners with and without Developmental Language Disorder. Journal of Speech, Language, and Hearing Research.

[R14] Durant K, Peña E, Peña A, Bedore LM, Muñoz MR (2019b). Not all nonverbal tasks are equally nonverbal: Comparing two tasks in bilingual kindergartners with and without Developmental Language Disorder. Journal of Speech, Language, and Hearing Research.

[R15] Earle FS, Gallinat EL, Grela BG, Lehto A, Spaulding TJ (2017). Empirical implications of matching children with specific language impairment to children with typical development on nonverbal IQ. Journal of Learning Disabilities.

[R16] Ebert KD, Kohnert K (2011). Sustained attention in children with primary language impairment: A meta-analysis. Journal of Speech, Language, and Hearing Research: JSLHR.

[R17] Ebert KD, Lee H (2024). Individual predictors of language treatment response in children with developmental language disorder: A systematic review. Journal of Speech, Language, and Hearing Research.

[R18] Ebert KD, Rak D, Slawny CM, Fogg L (2019). Attention in bilingual children with developmental language disorder. Journal of Speech, Language, and Hearing Research.

[R19] Elo S, Kyngäs H (2008). The qualitative content analysis process. Journal of Advanced Nursing.

[R20] Esterman M, Rothlein D (2019). Models of sustained attention. Current Opinion in Psychology.

[R21] Frost R, Armstrong BC, Christiansen MH (2019). Statistical learning research: A critical review and possible new directions. Psychological Bulletin.

[R22] Gallinat E, Spaulding TJ (2014). Differences in the performance of children with specific language impairment and their typically developing peers on nonverbal cognitive tests: A meta-analysis. Journal of Speech, Language, and Hearing Research: JSLHR.

[R23] Gerrig RJ, Banaji MR, Sternberg RJ (1994). Thinking and Problem Solving.

[R24] Kaufman AS, Kaufman NL (2004). Kaufman Brief Intelligence Test—Second Edition.

[R25] Krassowski E, Plante E (1997). IQ variability in children with SLI: Implications for use of cognitive referencing in determining SLI. Journal of Communication Disorders.

[R26] Miller CA, Gilbert E (2008). Comparison of performance on two nonverbal intelligence tests by adolescents with and without language impairment. Journal of Communication Disorders.

[R27] Nebraska Department of Education, Office of Special Education (2021). Determining Special Education Eligibility—Speech Language Impairment.

[R28] Norbury CF, Gooch D, Wray C, Baird G, Charman T, Simonoff E, Vamvakas G, Pickles A (2016). The impact of nonverbal ability on prevalence and clinical presentation of language disorder: Evidence from a population study. Journal of Child Psychology and Psychiatry, and Allied Disciplines.

[R29] Petersen SE, Posner MI (2012). The attention system of the human brain: 20 years after. Annual Review of Neuroscience.

[R30] Plante E (1998). Criteria for SLI. Journal of Speech, Language, and Hearing Research.

[R31] Posner MI, Rothbart MK (2007). Research on attention networks as a model for the integration of psychological science. Annual Review of Psychology.

[R32] Raven J, Raven J (2003). Handbook of nonverbal assessment.

[R33] Roid GH, Mille LJ, Pomplun M, Koch C (2013). Leiter International Performance Scale—Third Edition.

[R34] Saffran JR (2018). Statistical learning as a window into developmental disabilities. Journal of Neurodevelopmental Disorders.

[R35] Schapiro A, Turk-Browne N (2015). Brain Mapping.

[R36] Smolak E, McGregor KK, Arbisi-Kelm T, Eden N (2020). Sustained attention in developmental languag disorder and its relation to working memory and language. Journal of Speech, Language, and Hearing Research: JSLHR.

[R37] Stark RE, Tallal P (1981). Selection of children with specific language deficits. The Journal of Speech and Hearing Disorders.

[R38] Strand JF, Ray L, Dillman-Hasso NH, Villanueva J, Brown VA (2020). Understanding speech amid the jingle and jangle: Recommendations for improving measurement practices in listening effort research. Auditory Perception & Cognition.

[R39] Swisher L, Plante E (1993). Nonverbal IQ tests reflect different relations among skills for specifically language-impaired and normal children: Brief report. Journal of Communication Disorders.

[R40] Tang Y-Y, Hölzel BK, Posner MI (2015). The neuroscience of mindfulness meditation. Nature Reviews Neuroscience.

[R41] Ullman MT, Pierpont EI (2005). Specific Language Impairment is not Specific to Language: The Procedural Deficit Hypothesis. Cortex.

[R42] Wechsler D (2011). Wechsler Abbreviated Scale of Intelligence—Second Edition.

[R43] Wechsler D (2012). Wechsler Preschool and Primary Scale of Intelligence—Fourth Edition.

[R44] Wechsler D (2014). Wechsler Intelligence Scale for Children—Fifth Edition.

